# E-learning programs in oncology: a nationwide experience from 2005 to 2014

**DOI:** 10.1186/s13104-017-2372-8

**Published:** 2017-01-13

**Authors:** Jan Degerfält, Staffan Sjöstedt, Per Fransson, Elisabeth Kjellén, Mads U. Werner

**Affiliations:** 1 Department of Clinical Sciences, Division of Oncology and Pathology, Lund University, Barngatan 2B, 221 85 Lund, Sweden; 2 LäraNära AB, Drottninggatan 11, 252 21 Helsingborg, Sweden; 3 Department of Nursing, Umeå University, 901 87 Umeå, Sweden; 4 Multidisciplinary Pain Center 7612, Neuroscience Center, Rigshospitalet, Copenhagen University Hospital, Blegdamsvej 9, 2100 Copenhagen O, Denmark

**Keywords:** Cytostatic agents, Distance education, Medical oncology, Pain, Personal satisfaction, Professional education, Quality improvement, Questionnaires, Radiation oncology

## Abstract

**Background:**

E-learning is an established concept in oncological education and training. However, there seems to be a scarcity of long-term assessments of E-learning programs in oncology vis-á-vis their structural management and didactic value. This study presents descriptive, nationwide data from 2005 to 2014. E-learning oncology programs in chemotherapy, general oncology, pain management, palliative care, psycho-social-oncology, and radiotherapy, were reviewed from our databases. Questionnaires of self-perceived didactic value of the programs were examined 2008–2014.

**Results:**

The total number of trainees were 4693, allocated to 3889 individuals. The trainees included medical doctors (MDs; n = 759), registered nurses (RNs; n = 2359), radiation therapy technologists (n = 642), and, social and health care assistants (SHCAs; n = 933). The E-learning covered 29 different program classifications, comprising 731 recorded presentations, and covering 438 themes. A total of 490 programs were completed by the trainees. The European Credit Transfer and Accumulation System (ECTS; 1 ECTS point equals 0.60 US College Credit Hours) points varied across the educational programs from 0.7 to 30.0, corresponding to a duration of full-time studies ranging between 15 to 900 h (0.4–24 weeks) per program. The total number of ECTS points for the trainee cohort, was 20,000 corresponding to 530,000 full-time academic hours or 324.0 standard academic working years. The overall drop-out rate, across professions and programs, was 10.6% (499/4693). The lowest drop-out rate was seen for RNs (4.3%; *P* < 0.0001). Self-reported evaluation questionnaires (2008–2014) were completed by 72.1% (2642/3666) of the trainees. The programs were overall rated, on a 5-categorical scale (5 = excellent; 1 = very inferior), as excellent by 68.6% (MDs: 64.9%; RNs: 66.8%; SHCAs: 77.7%) and as good by 30.6% (MDs: 34.5%; RNs: 32.4%; SHCAs: 21.5%) of the responders.

**Conclusions:**

This descriptive study, performed in a lengthy timeframe, presents high-volume data from multi-professional, oncological E-learning programs. While the E-learning paradigm, across professions, seems to have been well received, it is imperative that prospective studies, benchmarking against traditional training methods, are carried out, examining the hypothesized didactic value of our E-programs.

**Electronic supplementary material:**

The online version of this article (doi:10.1186/s13104-017-2372-8) contains supplementary material, which is available to authorized users.

## Background

E-learning, also called computer-based learning, online learning or web-based learning, is a ubiquitously used technology in higher education [1–3]. E-learning comprises internet-based, interactive and asynchronous teaching and learning tools. A number of open universities, some even ‘mega-universities’ with more than 100,000 students, have adopted these as standard education techniques. US National Center for Educational Statistics, in the 2011 report, states that 30% of all students with bachelor’s degrees (n = 860,000) were enrolled in distance education courses with 75% of these taking their entire postgraduate program online [4, 5]. Probably, more than 80% of U.S. doctoral/research institutions have some form of online offering, either courses or full programs [6].

A number of advantages of E-learning programs, vis-á-vis traditional education programs, have been hypothesized: uncoupling of education from time and place, standardization of instruction and assessment, ease of documentation of learner behavior, student control of the education experience and increased educational cost-effectiveness [7]. A recent meta-analysis [8] indicates that no-intervention studies of internet-based learning, using pre- and post-tests, demonstrated augmented effectiveness in relation to acquisition of knowledge (factual or conceptual understanding) and skills. However, in studies comparing internet-based learning with non-internet-based traditional methods there only seems to be a small educational benefit from internet-based learning [8, 9]: a likely explanation is the large heterogeneity and variance in the data, characterizing the studies.

Oncology is indeed a challenging subject matter not only for any trainee, but also for the educator [2]. The E-learning programs may overcome some of the difficulties seen with traditional learning programs by allowing flexibility in time, place, and pace, for the clinically working trainee and educator [10]. There is a paucity of detailed, descriptive long-term data regarding management and outcome of E-learning programs. This study presents outcome data from our E-learning programs in oncology: chemotherapy; general oncology; pain management; palliative care; psycho-social-oncology; and radiotherapy, covering a span of 10 years, and including various health-care professions: medical doctors; nurses; radiation therapy technologists; and social and health care assistants.

## Methods

### Ethics

The E-programs and the associated data-bases complied with Swedish laws and regulations stipulated in the Personal Data Act (1998:204), aiming to prevent the violation of personal integrity in the processing of personal data [11]. Since the study was retrospective and registry-based, an application to the ethical committee was not considered necessary.

### Organization behind the E-learning programs

The organization behind the E-learning programs (E-programs) was established in 2002 by the authors (JD, SS), affiliated to the Department of Oncology, Clinical Sciences, Lund University. Initially, the E-programs were made and driven by local specialists: oncologists, radiation therapy technologists, medical physicists and anesthesiologists. After some years of benchmarking, both regional and national teaching resources were successively recruited into the E-programs. The supplier of the E-programs was the Department of Oncology, University Hospital of the Southern Region, in collaboration with the Medical Faculty at Lund University.

### Commissioning parties

The primary commissioning parties were local (n = 17), or regional, university-based (n = 7), oncological departments in Sweden.

### Financial aspects

The trainees’ tuition fees were reimbursed by their affiliated oncological departments, in turn recompensed by the respective County Council [Sweden comprises 21 County Councils (across six Health Care Regions) responsible for financing and providing health care managed by the Ministry of Health and Social Affairs]. In 7 out of 20 E-programs, co-financing with the Swedish Medical Association, the National Board of Health and Welfare, the Association of County Councils, and the National Agency for Higher Vocational Education, were agreed upon.

### Structure of the education

**Fig. 1:**
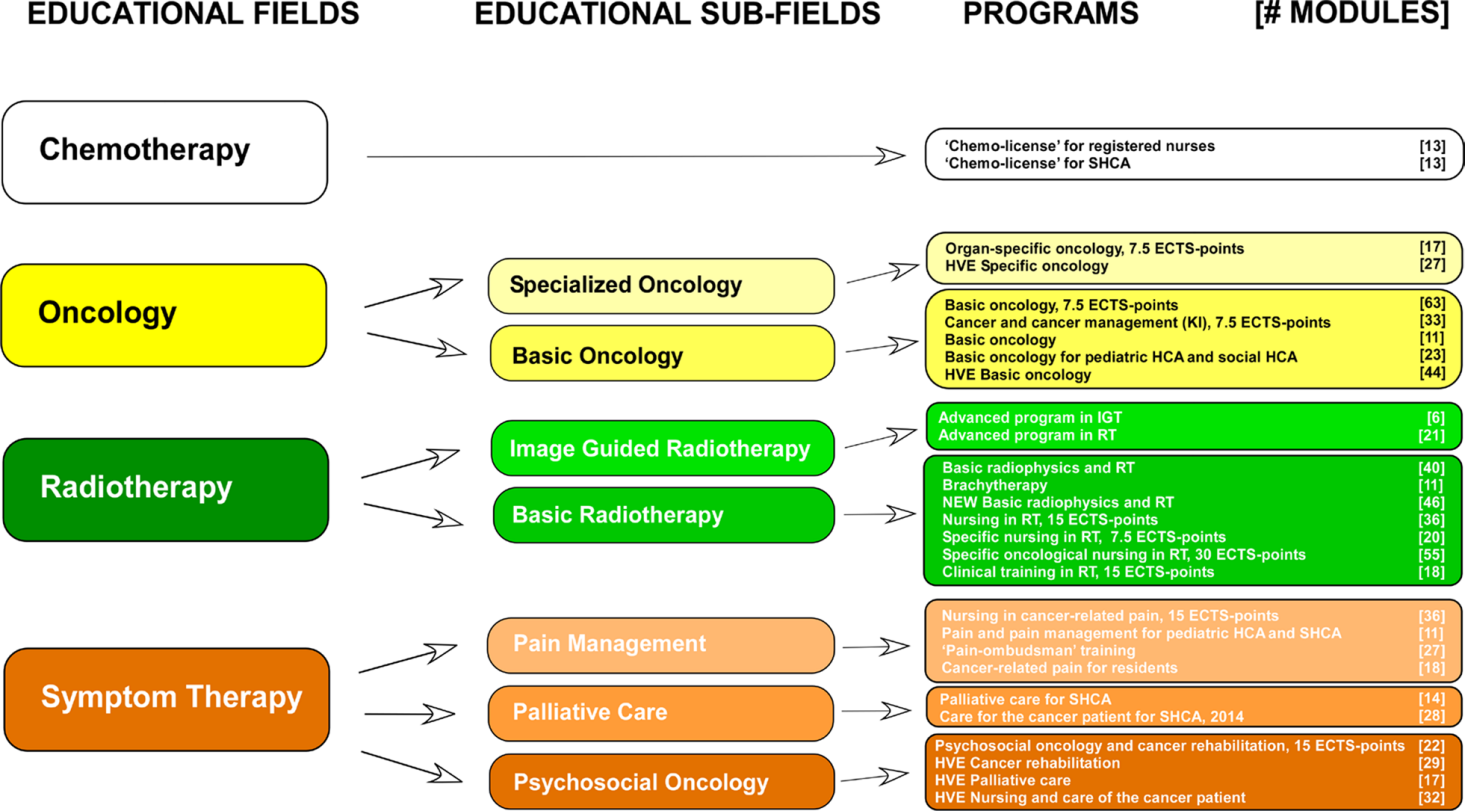
*Educational Fields*, *Educational Sub*-*Fields*, *Programs* and number of *Modules* in each E-program. *Programs* are indicated by name, number of ECTS-points (European Credit Transfer and Accumulation System points or equivalents; 1 ECTS-point corresponds to 25–30 h of study, equivalent to one Swedish University College point or 0.60 US College Credit Hours). The total number of *Modules* in the *Programs* are 731. *HCA* health care assistants, *HVE* higher vocational education, *IGRT* image-guided radiotherapy, *RT* radiotherapy, *SHCA* social and health care assistants

The structure included four hierarchical levels: *Educational Fields*, *Educational Sub*-*Fields*, *Programs*, and *Modules* **(**Fig. 1). The *Educational Fields* covered Chemotherapy, Oncology, Radiotherapy and Symptom Therapy. The *Educational Sub*-*Fields* covered for the *Educational Fields*, Oncology: Basic Oncology, Specialized Oncology; Radiotherapy: Basic Radiotherapy, Image Guided Radiotherapy; and for Symptom Therapy: Palliative Care; Pain Management; Psychosocial Oncology. The *Educational Field*, Chemotherapy, however, did not accommodate any *Educational Sub*-*Field* (Fig. 1).

The *Programs*, included a categorization of topics based on trainees’ profession, e.g., Basic Oncology for social and health care assistants, Radiotherapy for registered nurses, and, Radiotherapy for medical doctors. The trainees’ professions and required proficiency levels across *Educational Sub*-*Fields*, and the duration of individual E-programs, are presented in Table 1. A detailed description of an E-program, representative in Radiotherapy for medical residents in oncology is illustrated in Table 2. Illustrations of E-programs from each of the four *Educational Fields* are presented in Table 3. Finally, *Modules* constitute the basic educational building blocks, usually aggregated in a number of themes (Table 2).

**Table 1:** The *Educational Fields*, trainees’ health care professions, trainees’ proficiency levels, *Program* duration and ECTS-points [European Credit Transfer and Accumulation System or equivalent scoring system (1 ECTS-point corresponds to 25–30 h of study)], start year of the *Programs* and number of *Programs* given (n = 490)

Educational Fields	Educational sub-fields	Profession	Proficiency level	Program duration	ECTS-point	Start year	No. programs given
Chemotherapy		RN, SHCA	BAS	1 week	VC	2005	154
Oncology	Specialized oncology	RN, SHCA	BAS	5 weeks	7.5	2007	27
	Basic oncology	RN, SHCA	BAS	5 weeks	7.5	2007	62
Radiotherapy	Image guided radiotherapy	RT	EXP	1 week	VC	2010	23
	Basic radiotherapy	MD, RN, RTT, SPEC	BAS, EXP, RES	6–20 weeks	9–30^a^	2005	72
Symptom therapy	Palliative care	SHCA	BAS	5–10 weeks	7.5–15	2012	18
	Pain management	HCA, MD, RN, SHCA	BAS, EXP, RES, SPEC	5–10 weeks	7.5–15	2005	131
	Psychosocial oncology	RN, SHCA	BAS	1–10 weeks	0.7–15^a^	2010	3

^a^Includes additional residence programs and vocational clinical programs; *APRN* advanced practice registered nurses, *BAS* basic level, *EXP* experienced level, *HCA* pediatric health care assistants, *MD* medical doctors, *RES* residents, *RN* registered nurses, *RTT* radiation therapy technologists, *SHCA* social and health care assistants, *SPEC* specialists (MD), *VC* vocational, clinical training

**Table 2:** A representative sample of an E-program: “Basic radiophysics and radiotherapy for residents in oncology”

*Educational Field*	
Radiotherapy	
*Educational sub-field*	
Basic radiotherapy	
*Program*	
Basic radiophysics and radiotherapy for residents in oncology	
*Modules arranged in themes*	
Radiation physics	
Radiation and atoms	Radiation from radioactive decay and artificial radiation
Interactions between radiation and materials	Contrast, kV vs. MV
Accelerators	Equipment for radiation therapy with low energy X-rays
The photon radiation field	Measuring radiation doses
Questions in radiological physics	History of Radiotherapy
Computed tomography in radiation therapy	Course seminar no 1
Treatment planning and fractionation	
Biologically Effective Dose	Target
Immobilisation Aids	Patient positioning
External radiotherapy techniques	Dose verification in clinical practice
Treatment Planning	Dose-volume histograms (DVH)
History of ICRU and Radiotherapy	PET in Radiotherapy
Palliative Radiotherapy	Introduction to brachytherapy
Course seminar no 2	
Optimisation	
Gold Anchor	Optimisation
Late complications and RTOG-score	Secondary cancers in radiotherapy
Re-irradiation	Stereotactic Body Radiotherapy (SBRT)
Proton treatment	Radiation risks for personnel and the public
Radiation protection for external radiation therapy	Course seminar no 3
*Groups*	
Duration: 9 month part-time, corresponding to 9 ECTS	
2–3 groups/year	
6 tutors/group	
3–4 seminars/group	
Example: Group nr. 28. Jan.–Sept. 2014	
12 trainees: Lund 3, Stockholm 3, Kalmar 2, Göteborg 1, Jönköping 1, Sundsvall 1, Umeå 1	
3 seminar days in March, May and Sept., 2014

**Table 3:** Various samples of E-programs from each Educational Field in regard to the trainees’ profession, scheduled Program duration, maximum allowed study time, the number of physical meetings and the duration of each meeting

Educational Fields	Program	Profession	Duration	Stipulated study time	Physical meetings	Duration
Chemotherapy	‘Chemo-license’	RN	25 h	5 weeks	1	3 h
	‘Chemo-license’	HCA	25 h	5 weeks	1	3 h
Oncology	Organ-specific oncology	RN	5 weeks	5 weeks	1	4 h
	Basic oncology	RN	5 weeks	5 weeks	1	1 day
	HVE Basic oncology	SHCA	5 weeks	10 weeks	2	2 days
Radiotherapy	Basic radio physics and RT	MD (residents)	10-15 days	9 mo	2	1 day
	Specific oncological nursing in RT	RN	20 weeks	20 weeks	3	2 days
	Brachytherapy	RN	15 h	no limit	0	–
Symptom therapy	Nursing in cancer-related pain	RN	10 weeks	20 weeks	4	1.5 days
	‘Pain-ombudsman’ training	RN, MD	20 h	3 mo	1	1 day
	Psychosocial oncology and cancer rehabilitation	RN	10 weeks	20 weeks	4	1.5 days

*HVE* higher vocational education, *MD* medical doctors, *RN* registered nurses, *RT* radiotherapy, *SHCA* social and health care assistants

#### Professions

Health care professions included were, registered nurses (RNs), medical doctors (MDs), social and health care assistants (SHCAs; 7% of SHCAs belonged to administrative personnel), and radiation therapy technologists (RTTs). The trainees’ proficiencies were classified into basic, specialist-in-training or specialist levels (Table 1).

#### Recruitment

Three different recruitment paths were utilized. Most commonly an educational contract between the trainee’s oncological department, mainly university-based departments, and the supplier of the E-program, was established, stipulating the educational requirements. Recruitment by advertisements in journals, flyers or web-sites for health care professionals were also utilized. A substantial number of trainees were also recruited by word-of-mouth and selected from waiting lists. Trainees were affiliated to anesthesiological, medical, oncological or surgical departments.

### Tutors and producers

#### Professions

Tutors were senior ranking clinical oncologists and anesthesiologists, many with an academic affiliation. Producers of the educational material included specialists from various disciplines: advanced practice registered nurses, medical doctors, physicists and psychologists, almost all with senior clinical background in addition to an academic affiliation.

#### Recruitment

Tutors and producers were recruited nationwide from professional, academic networks [JD (palliative care; psychosocial oncology; radiotherapy), EK (oncology; radiotherapy), MW (pain management)].

### Basic structure of the module

#### Design

The design of the *Module* was based on theory-derived principles of educational practice, following the recommendations on web-based learning by Cook and Dupras [12]. The goals and objectives of each *Module* were pre-specified and presented at an early stage to the trainee, the IT-platform was successively tailored to the needs of the trainees and the tutors, appropriate multimedia and hyperlinks were used and an active learning-approach was encouraged (self-assessment, reflection, self-directed learning, problem-based learning, learner interaction, and feedback). The IT-platform underwent substantial changes and improvements during 2005–2014, reflecting the general technical progress in the IT-field. To address the need for real-life, clinical problems, case-based training scenarios were incorporated into the *Modules*.

#### Educational material

The specialists either by themselves, depending on their IT-proficiency, or, in collaboration with the IT-technical staff (SS, JD) produced lectures, usually in the format of multimedia presentations (PowerPoint with voiceover speech). Since 2006 a web-based “authors’ tool box” facilitating the production has been available for import of images and audio-files. The *Module* is based on one or more presentations (Table 2), Additional file 1 (e.g., scientific literature, guidelines, images, animations), links to relevant web-sites, web-based tests intended to establish performance status (MCQ, short essays) and the trainees’ web-based self-evaluation of the training quality of the *Module*. In addition conventional or online education materials like textbook chapters sometimes were employed by the tutors. From 2005 to 2011 the educational material was CD-based, but thereafter online log-in procedures were employed.

#### Educational objectives

Where a core curriculum stipulated by national consensus and based on national guidelines, were available, this was applied for each respective *Educational Field*, e.g., Radiotherapy, and correspondingly for each hierarchical level, i.e., *Educational Sub*-*Field*, *Program* and *Module*.

#### E-based interactivity

Interactivity was considered essential in encouraging the trainee’s active learning process [12], including self-assessments, self-directed learning often based on problem-solving issues and interaction with the tutor. The trainee’s activity on the technical platform was logged, and information on latest logins and lectures viewed, was available for the tutors. In order to augment self-assessment and self-directed learning a procedure called *self*-*evaluation* was used in essay exercises. After submission of the essay the trainee automatically received a complete essay report pre-fabricated by the tutor, and was then asked to re-submit the report after considering necessary changes from the original response, at the discretion of the trainee. The tutor then reviewed the re-submission and gave an individualized feedback on the essay report to the trainee. This simple *self*-*evaluation* formative measure seemed to increase the didactical value both for the trainee and the tutor, in addition to decreasing the workload of the tutor. Further, it also helped to pin down the learning objectives, facilitating the awareness of the trainee that the core goals had been achieved. But most importantly, it gave the trainee a learning opportunity and helped to the tutor’s recognition of didactic misunderstandings. E-based interactivity between fellow-trainees was based on asynchronous fora.

#### Physical meetings

During the first years of the E-programs a requirement for physical meetings, vis-á-vis virtual meetings, became apparent, and, we, empirically incorporated 2–3 compulsory meetings of 1½-days duration for every 4–6 months of participation in the E-program. Furthermore, it was required that the trainees completed all modules and seminars in order to get approval of the course. The rationales behind these meetings were based on practical, didactic and social aspects. *First*, when the E-programs were initiated 2003–2005, conventional learning methods, were still considered “the gold standard”, at least across the targeted professions and the age groups, and therefore a noticeable demand for physical meeting existed. *Second*, although the general acceptance of E-learning methods has increased dramatically during the last decades [13], it has been our experience that the physical gatherings are still justified since the trainees’ active learning process is stimulated. *Third*, needless to say, it consolidates the social networking, important since trainees often may live at considerable distance, sometimes up to thousand miles from each other. *Fourth*, a number of studies seem to indicate that the drop-out rates in E-programs decrease by use of physical meetings.

#### Technical aspects

An extended E-support for trainees and tutors was instituted from the beginning of the E-programs, and was quickly considered a prerequisite for successful implementation. A number of the trainees the first years generally demonstrated a lack of prowess and routine in IT-issues, requiring basic support beyond the supplied instruction manuals. During 2005–2011 the bulk of the programs, as previously mentioned, were CD-based requiring installation routines sometimes associated with technical incompatibility problems across systems and drivers. An IT-engineer (SS) and a highly qualified technician (JD) were at all times available, particularly important, during server-malfunctioning problems. The maintenance, development and improvements of the platform were by the engineer and an IT-assistant.

### Outcomes

#### Learning objectives

After the completion of each *Module* the trainee evaluated how well the training had achieved its objective by a number of standardized questions including simple categorical rating scales. At completion of the program the trainee was also asked to rate qualitative aspects of the education material. These evaluations were continuously used by the tutors as an important feed-back mechanism on the didactic quality of the E-programs.

#### Other

Drop-out rates, across professions and programs, were calculated from our database as:

$${\text{drop out rate}} \% = 100 \times \frac{{{\text{number registered}} - {\text{number completed}}}}{\text{number registered}}$$

### Data collection

Data were collected from our central database, from January 1, 2005 to December 31, 2014, containing information on participants’ initials, profession, zip-code, program affiliation (entry and completion date), tutor affiliation and response to questionnaire on learning objectives. In addition, information regarding the tutors’ and producers’ profession, academic education and program affiliation was retrieved from the database. A technical database was accessed analyzing the completed number of *Programs* and *Modules*, and, the technical structure of the *Modules* in regard to number of frames, the duration of the oral presentation, the cumulated frame time for the available lectures and use of video sequences.

#### Statistical methods

Normality of continuous data was analyzed by the Kolmogorov–Smirnov test and visual inspection of residual plots, and, un-paired comparisons were by a *t* test or by Mann–Whitney test, as appropriate. Categorical data were analyzed by Chi-squared test or Fisher’s exact test, as appropriate. Linear univariate regression analysis was by calculation of Pearson’s correlation coefficient. Statistical evaluations were by MedCalc Software (v. 12.07.0.0; Mariakerke, Belgium). Data, subjected to multiple comparisons, were corrected by the Bonferroni method, in order to decrease the likelihood of type I errors. Statistical significance was assigned at *P* < 0.05. Parametric and non-parametric data are presented as mean [95% confidence interval (CI)] or median (25–75% interquartile range [IQR]), respectively.

## Results

### Educational structure

An overview of the educational structure is presented in Table 1 including: the *Educational Fields*; the *Educational Sub-Fields*; the trainees’ health care professions; the trainees’ proficiency levels; the *Program* duration; corresponding ECTS-points [European Credit Transfer and Accumulation System points (one ECTS-point corresponds to 25–30 h of full-time study, equivalent to one Swedish University College point or 0.60 US College Credit Hours)]; starting year of the *Program*; and the number of completed *Programs*. The ECTS-points varied across the educational *Programs* from 0.7 to 30.0 ECTS-points, corresponding to a duration of full-time studies ranging between 15 to 900 h (0.4–24 weeks) per *Program*.

The 29 different *Program* classifications, contained a total of 731 *Modules* covering 438 themes (Fig. 1). The trainees completed a total of 490 *Programs*. Each *Module* presentation contained in median 18 frames (IQR 11; 26) with an oral presentation time of each frame of 0.68 min (IQR 0.38; 1.12), corresponding to a total time for each presentation of 12 min (7.48; 17.68). The cumulated frame time for all available lectures was 10,605 min (177 h). Video presentations were available in 30 *Modules* with a total number of video sequences of 64.

### Demographics

#### Trainees

**Fig. 2:**
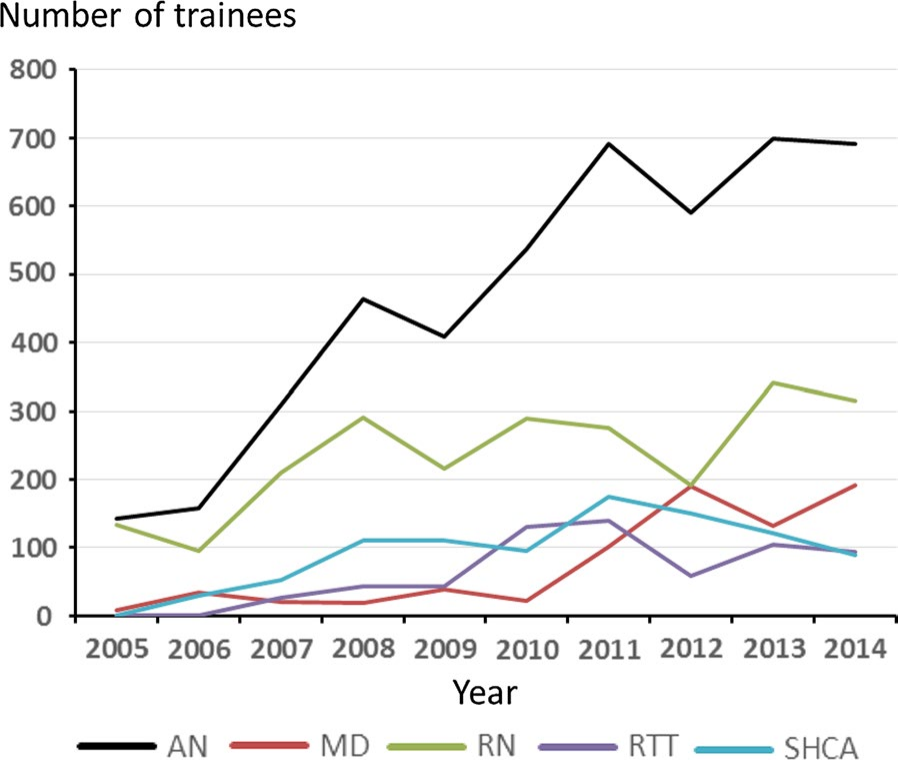
The annual number of trainees (AN; total number = 4693) 2005–2014 across professions: registered nurses (RN; total number = 2359); radiation therapy technologists (RTT; total number = 642); medical doctors (MD; n = 759); and, social and health care assistants (SHCA; total number = 933). The number deviate from the total number of individual trainees (n = 3889), due to 20.7% of trainees’ participation in more than one *Program*

**Fig. 3:**
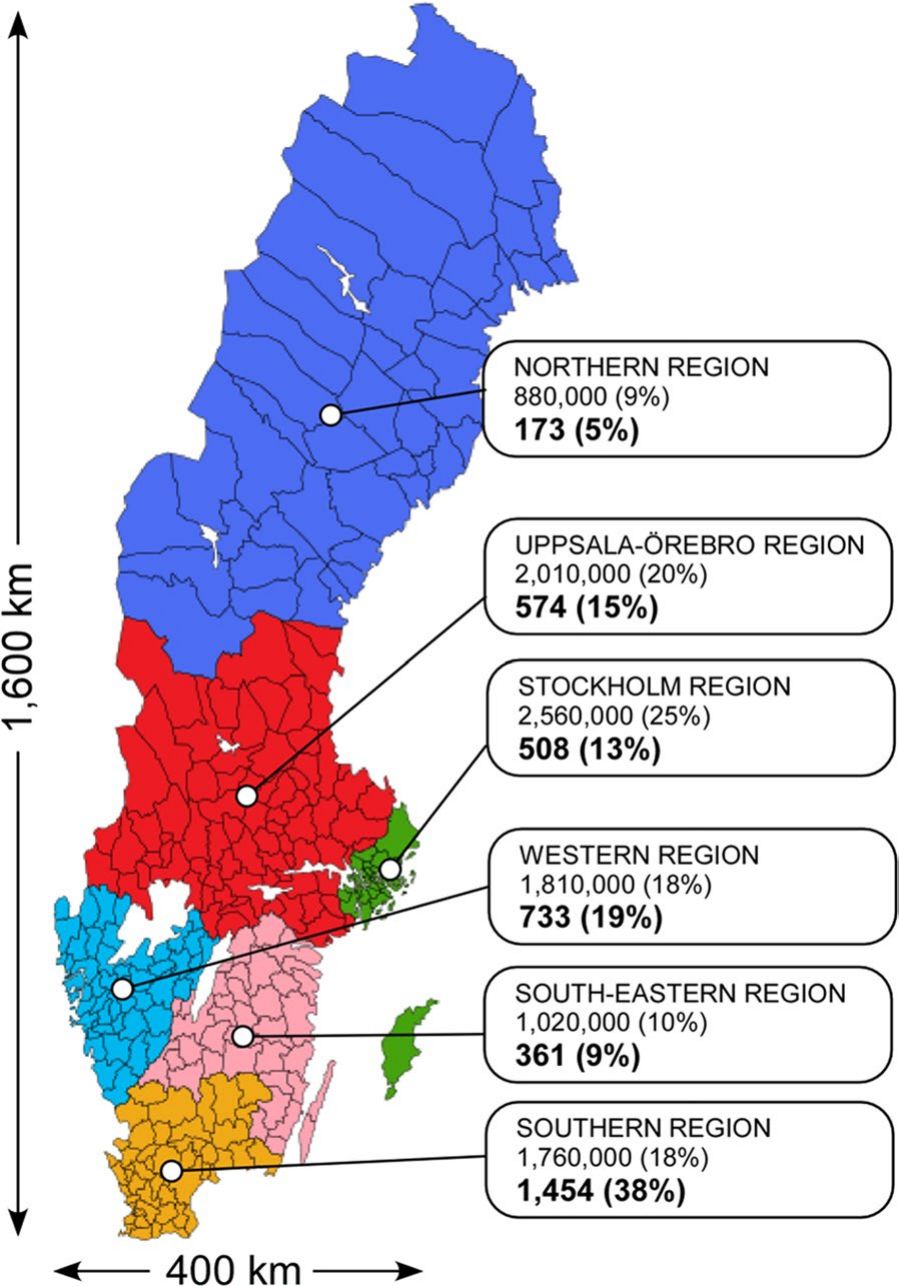
The six health care regions in Sweden with populations (%) as per December 31, 2014, and the distribution of number of trainees (%) in each health care region, from 2005 to 2014. Total population of Sweden, at this time point, was 9750,000, and total number of trainees was 3889 (33 subjects had their residence outside Sweden; map accessed at https://sv.wikipedia.org/wiki/Sjukv%C3%A5rdsregion; May 6, 2015)

**Fig. 4:**
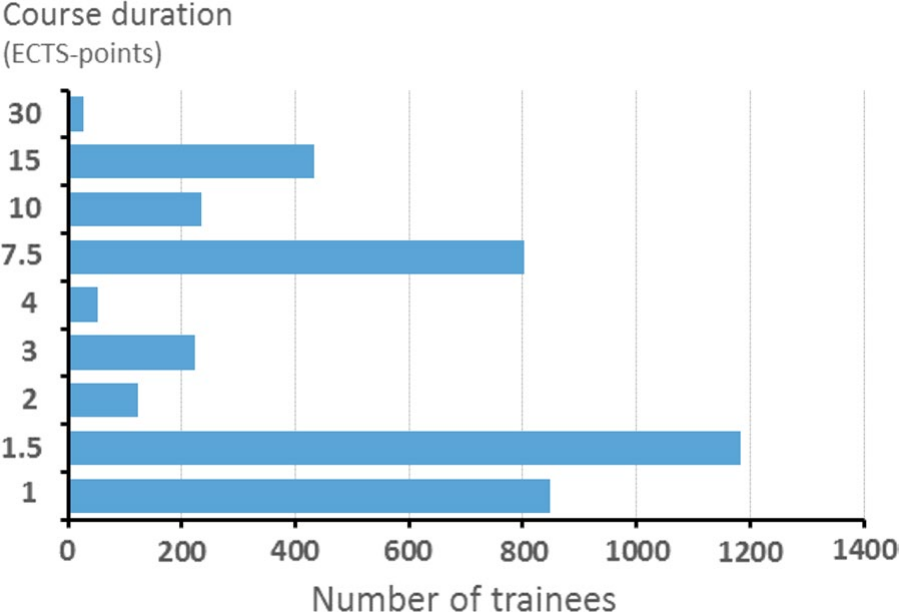
The relationship between number of trainees (n = 3926) and the duration of courses (ECTS-points; 1 ECTS-point corresponds to 25–30 h of full-time study, equivalent to one Swedish University College point or 0.60 US College Credit Hours) from 2005 to 2014

The annual number of trainees, across professions, from 2005 to 2014, are presented in Fig. 2. A total of 4693 trainees completed the *Programs*, while the total number of individuals were 3889, since 20.7% of the trainees participated in more than one *Program*. The trainees’ geographical distribution across the six Swedish health care regions, is illustrated in Fig. 3. The bimodal relationship between number of trainees (n = 3926) and duration of courses (ECTS-points) is indicated in Fig. 4. The percentage of trainees attending courses corresponding to ≤4 ECTS-points was 61.9%, and to ≥7.5 ECTS-points 38.1%. The total number of ECTS-points for the trainee-cohort was 19,438, corresponding to 534,545 full-time academic hours, equaling 324.0 standard working years (Joint Costing and Pricing Steering Group for Higher Education Institutions, U.K.) [14]. The distribution of professions of the trainees, across *Educational Fields*, is illustrated in Table 4.

**Table 4:** Distribution of professions of the trainees in regard to *Educational Fields* (n = 4693)

Educational Fields	MD	RN	SHCA	RTT
Chemotherapy	0	1200	280	0
Oncology	0	599	306	0
Radiotherapy	241	431	0	642
Symptom Therapy	518	170	347	0
Total	759	2359	933	642

Data are skewed according to professions (Chi-squared test, *P* < 0.0001, contingency coefficient = 0.540)

*MD* medical doctors, *RN* registered nurses, *RTT* radiation therapy technologists, *SHCA* social and health care assistants

#### Tutors and producers

The professions of the tutors (n = 78), the producers of the educational material (n = 82) and the combined producer-tutors (n = 34), are illustrated in Table 5. The number of individuals with an academic affiliation (i.e., a PhD-degree) among MDs, RNs and ʻother professionsʼ were for tutors 9/28 (32.1%), 1/35 (2.9%) and 10/15 (66.7%), respectively. Corresponding numbers for producers were 22/29 (75.9%), 3/22 (13.6%) and 18/31 (58.1%). Across professions, comparing tutors with producers, a significantly increased proportion of individuals with academic affiliation was observed for tutors [*P* = 0.0001 (Chi-squared test)], most evident for MDs [75.9 vs. 32.1%; *P* < 0.003 (Fisher’s exact test)]. A significantly higher proportion of MDs and ʻother professionsʼ had an academic affiliation compared to RNs [*P* < 0.003 (Fisher’s exact test)]. The numerical higher proportion with academic affiliation for ʻother professionsʼ compared with MDs (Table 5) did not reach significance for tutors or for producers [*P* = 0.052 and *P* = 0.18, respectively (Fisher’s exact test).]

**Table 5:** Distribution of professions of the tutors, the producers (producing education material) and the combined producer-tutors

Professsion	n	+ Tutor	− Tutor	+ Producer	− Producer	+ Producer + Tutor	PhD
MD	49	28	21	29	20	8	26
RN	44	35	9	22	22	13	4
Other	34	15	19	31	3	13	20
Total	127	78	49	82	45	34	50

The number of PhDs for each profession indicate the academic affiliation. Medical doctors (MD) and registered nurses (RN) comprised 53.1 and 9.1%, respectively. Other professions (26.8%) included a legal counselor (n = 1), dental hygienist (n = 1), dieticians (n = 2), high school teacher (n = 1), pharmacist (n = 1), physiotherapists (n = 3), psychologist (n = 1), radiophysicists (n = 19), medical researchers (n = 3), and, social and health care assistants (n = 2)

### Outcomes

#### Self-reported evaluation of learning objectives

From January 1, 2008 to December 31, 2014 evaluations, based on a written questionnaire, were delivered to the trainees upon completion of the program. Questionnaires were available and evaluated from 72.1% (2642/3666) of the trainees. Fully completed questionnaires, were obtained from 96.5% [mean (CI: across programs); 94.2–98.7%] of the respondents. Below statistics are presented for the individual 10 questions (A–J; italic):

- A. How would you rate the program as a whole?

The *Programs* were, across professions, overall rated as excellent by 68.6% and as good by 30.6% of the responders. SHCAs demonstrated significantly higher overall ratings of the *Programs* than RNs and MDs (Tables 6, 7).

**Table 6:** Tabular data for self-reported evaluation of learning objectives across professions (total number of trainees for each profession)

Profession	Ratings				
	Excellent	Good	Neither good nor inferior	Inferior	Very inferior
A. How would you rate the program as a whole?					
MD (365)	237 (64.9%)	126 (34.5%)	1 (0.3%)	0 (0.0%)	1 (0.3%)
RN (1765)	1179 (66.8%)	572 (32.4%)	15 (0.8%)	0 (0.0%)	0 (0.0%)
SHCA (369)	369 (77.7%)	102 (21.5%)	4 (0.8%)	0 (0.0%)	0 (0.0%)
ADM (27)	27 (77.1%)	8 (22.9%)	0 (0.0%)	0 (0.0%)	0 (0.0%)
Total (2641)	1812 (68.6%)	808 (30.6%)	20 (0.8%)	0 (0.0%)	1 (0.0%)

Profession	Ratings				
	To a very high degree	To a high degree	Neither to a high degree nor a low degree	To a low degree	To a very low degree
B. Will you be able to use what you learned in everyday clinical practice?					
MD (364)	120 (33.0%)	232 (63.7%)	11 (3.0%)	1 (0.3%)	0 (0.0%)
RN (1765)	867 (49.1%)	799 (45.3%)	87 (4.9%)	9 (0.5%)	3 (0.2%)
SHCA (476)	199 (41.8%)	247 (51.9%)	29 (6.1%)	1 (0.2%)	0 (0.0%)
ADM (35)	6 (17.1%)	22 (62.9%)	6 (17.1%)	1 (2.9%)	0 (0.0%)
Total (2640)	1192 (45.2%)	1300 (49.2%)	133 (5.0%)	12 (0.5%)	3 (0.1%)

Profession	Ratings				
	To a very high degree	To a high degree	Neither to a high degree nor a low degree	To a low degree	To a very low degree
C. Would you recommend the program to a colleague in a similar situation as yours?					
MD (365)	221 (60.5%)	130 (35.6%)	13 (3.6%)	1 (0.3%)	0 (0.0%)
RN (1763)	1233 (69.9%)	467 (26.5%)	60 (3.4%)	3 (0.2%)	0 (0.0%)
SHCA (476)	357 (75.0%)	102 (21.4%)	14 (2.9%)	2 (0.4%)	1 (0.2%)
ADM (35)	22 (62.9%)	10 (28.6%)	3 (8.6%)	0 (0.0%)	0 (0.0%)
Total (2639)	1833 (69.5%)	709 (26.6%)	90 (3.4%)	6 (0.2%)	1 (0.0%)

Profession	Ratings				
	Very high	High	Appropriate	Low	Very low
D. How did you experience the workload during the program?					
MD (364)	14 (3.8%)	153 (42.0%)	195 (53.6%)	2 (0.5%)	0 (0.0%)
RN (1749)	115 (6.6%)	472 (27.0%)	1120 (64.0%)	36 (2.1%)	6 (0.3%)
SHCA (471)	43 (9.1%)	163 (34.6%)	251 (53.3%)	10 (2.1%)	4 (0.8%)
ADM (35)	6 (17.1%)	18 (51.4%)	9 (25.7%)	2 (5.7%)	0 (0%)
Total (2619)	178 (6.8%)	806 (30.8%)	1575 (60.1%)	50 (1.9%)	10 (0.4%)

Profession	Ratings					
	Very High importance	High importance	Moderate importance	Low importance	Very low importance	Unimportant
E. Evaluate the importance of the recommended literature in the program.						
MD (341)	54 (15.8%)	126 (37.0%)	100 (29.3%)	44 (12.9%)	13 (3.8%)	4 (1.2%)
RN (1717)	513 (29.9%)	536 (31.2%)	403 (23.5%)	188 (10.9%)	58 (3.4%)	19 (1.1%)
SHCA (469)	137 (29.2%)	140 (29.9%)	96 (20.5%)	56 (11.9%)	30 (6.4%)	10 (2.1%)
ADM (34)	3 (8.8%)	14 (41.2%)	6 (17.6%)	6 (17.6%)	2 (5.9%)	3 (8.8%)
Total (2561)	707 (27.6%)	816 (31.9%)	605 (23.6%)	294 (11.5%)	103 (4.0%)	36 (1.4%)

Profession	Ratings					
	Very High importance	High importance	Moderate importance	Low importance	Very low importance	Unimportant
F. Evaluate the importance of the recommended scientific publications in the program.						
MD (334)	38 (11.4%)	100 (29.9%)	96 (28.7%)	64 (19.2%)	30 (9.0%)	6 (1.8%)
RN (1566)	112 (7.2%)	281 (17.9%)	411 (26.2%)	419 (26.8%)	215 (13.7%)	128 (8.2%)
SHCA (420)	66 (15.7%)	99 (23.6%)	118 (28.1%)	81 (19.3%)	48 (11.4%)	8 (1.9%)
ADM (33)	0 (0%)	10 (30.3%)	4 (12.1%)	8 (24.2%)	7 (21.2%)	4 (12.1%)
Total (2353)	216 (9.2%)	490 (20.8%)	629 (26.7%)	572 (24.3%)	300 (12.7%)	146 (6.2%)

Profession	Ratings					
	Very High importance	High importance	Moderate importance	Low importance	Very low importance	Unimportant
G. Evaluate the importance of the lectures in the program.						
MD (347)	164 (47.3%)	138 (39.8%)	37 (10.7%)	7 (2.0%)	0 (0%)	1 (0.3%)
RN (1739)	1116 (64.2%)	448 (25.8%)	114 (6.6%)	52 (3.0%)	3 (0.2%)	6 (0.3%)
SHCA (454)	312 (68.7%)	103 (22.7%)	18 (4.0%)	8 (1.8%)	5 (1.1%)	8 (2.8%)
ADM (35)	25 (71.4%)	7 (20.0%)	2 (5.7%)	1 (2.9%)	0 (0%)	0 (0%)
Total (2575)	1617 (62.8%)	696 (27.0%)	171 (6.6%)	68 (2.6%)	8 (0.3%)	15 (0.6%)

Profession	Ratings					
	Very High importance	High importance	Moderate importance	Low importance	Very low importance	Unimportant
H. Evaluate the importance of the links in the program.						
MD (341)	24 (7.0%)	90 (26.4%)	115 (33.7%)	76 (22.3%)	28 (8.2%)	8 (2.3%)
RN (1690)	251 (14.9%)	461 (27.3%)	474 (28.0%)	329 (19.5%)	133 (7.9%)	42 (2.5%)
SHCA (429)	134 (31.2%)	119 (27.7%)	94 (21.9%)	53 (12.4%)	19 (4.4%)	10 (2.3%)
ADM (35)	5 (14.3%)	10 (28.6%)	10 (28.6%)	6 (17.1%)	2 (5.7%)	2 (5.7%)
Total (2495)	414 (16.6%)	680 (27.3%)	693 (27.8%)	464 (18.6%)	182 (7.3%)	62 (2.5%)

Profession	Ratings					
	Very High importance	High importance	Moderate importance	Low importance	Very low importance	Unimportant
I. Evaluate the importance of the exercises and the feedback in the program.						
MD (344)	188 (54.7%)	113 (32.8%)	35 (10.2%)	7 (2.0%)	1 (0.3%)	0 (0%)
RN (1733)	864 (49.9%)	569 (32.8%)	194 (11.2%)	83 (4.8%)	18 (1.0%)	5 (0.3%)
SHCA (461)	281 (61.0%)	114 (24.7%)	42 (9.1%)	17 (3.7%)	5 (1.1%)	2 (0.4%)
ADM (34)	22 (64.8%)	8 (23.5%)	3 (8.8%)	1 (2.9%)	0 (0%)	0 (0%)
Total (2572)	1355 (52.7%)	804 (31.3%)	274 (10.7%)	108 (4.2%)	24 (0.9%)	7 (0.3%)

Profession	Ratings					
	Very High importance	High importance	Moderate importance	Low importance	Very low importance	Unimportant
J. Evaluate the importance of the physical meeting(s) in the program.						
MD (316)	127 (40.2%)	102 (32.3%)	49 (15.5%)	21 (6.6%)	9 (2.8%)	8 (2.5%)
RN (1640)	869 (53.0%)	483 (29.5%)	182 (11.1%)	81 (4.9%)	16 (1.0%)	9 (0.5%)
SHCA (438)	253 (57.8%)	126 (28.8%)	23 (5.3%)	24 (5.5%)	6 (1.4%)	6 (1.4%)
ADM (32)	16 (50.0%)	7 (21.9%)	5 (15.6%)	4 (12.5%)	0 (0%)	0 (0%)
Total (2426)	1265 (52.1%)	718 (39.6%)	259 (10.7%)	130 (5.4%)	31 (1.3%)	23 (0.9%)

Data are given as number (%) of trainees in each ordinal rating category. The data include ratings from 64.2 to 72.1% of the total number of trainees (n = 2353 to 2642) in the program 2008 to 2014 (n = 3666). For details regarding questions A–J cf. the text

*MD* medical doctors, *RN* registered nurses (in this table also includes radiation therapy technologists), *SHCA* social and health care assistants, *ADM* administrative personnel

**Table 7:** Statistical comparisons of self-reported, questionnaire-based outcomes across trainees’ professions (Chi-squared tests)

Questions	MDs vs.RNs	RNs vs. SHCA	MDs vs. SHCA
A. How would you rate the program as a whole?	1.00	0.003***	0.003***
B. Will you be able to use what you’ve learnt in everyday clinical practice?	0.003***	0.19	0.10
C. Would you recommend the program to a colleague in a similar situation as yours?	0.012*	0.82	0.003***
D. How did you experience the workload during the program?	0.03*	1.00	0.04*
E. Evaluate the importance of the recommended literature in the program	0.003***	1.00	0.003***
F. Evaluate the importance of the recommended scientific publications in the program	1.00	0.31	0.94
G. Evaluate the importance of the lectures in the program	0.003***	1.00	0.003***
H. Evaluate the importance of the links in the program	0.12	0.003***	0.003***
I. Evaluate the importance of the exercises and the feedback in the program	1.00	0.003***	0.58
J. Evaluate the importance of the physical meeting(s) in the program	0.39	1.00	0.21

The data include 64.2 to 72.0% of the total number of trainees (n = 2353 to 2641) in the program 2008 to 2014 (n = 3666). *P*-values are corrected by the Bonferroni correction for multiple comparisons (correction factor = 30; significant values in *italics*)

*MD* medical doctors, *RN* registered nurses, *SHCA* social and health care assistants

* *P* < 0.05; ** *P* < 0.01; *** *P* < 0.005

- B. Will you be able to use what you’ve learnt in everyday clinical practice?

The clinical applicability of the *Programs* was rated as “to a very high degree” of 45.2% and “to a high degree” of 49.2% of the responders. MDs demonstrated significantly lower ratings compared to RNs, while no differences compared to SHCAs were seen (Tables 6, 7).

- C. Would you recommend the program to a colleague in a similar situation as yours?

The recommendability of the *Programs* was rated as “to a very high degree” of 69.5% and “to a high degree” of 26.6% of the responders. Interestingly, RNs and SHCAs rated the recommendation value of the programs significantly higher than the MDs (Tables 6, 7).

- D. How did you experience the workload during the program?

The workload was experienced as “appropriate” to “very low” by 62.4% and as “very high” to “high” by 37.6%. Interestingly, the workload during the programs was perceived as relatively higher by the RNs and SHCAs, compared to MDs (Tables 6, 7).

- E. Evaluate the importance of the recommended literature in the program. F. Evaluate the importance of the recommended scientific publications in the program.

The importance of the recommended literature and the scientific publications (e.g., clinical studies, chapters from textbooks) was considered of “very high importance” to “high importance” by 44.8%, and of “moderate importance” by 25.2% of the responders. Both RNs and SHCAs evaluated the relative importance of the recommended literature significantly higher than the MDs (Tables 6, 7). In contrast, there were no statistical differences across professions in regard to the value of the recommended scientific publications (Tables 6, 7).

- G. Evaluate the importance of the lectures in the program.

Correspondingly, the importance of the recorded lectures was considered of “very high importance” to “high importance” by 89.8%, and of “moderate importance” by 6.6% of the responders. RNs and SHCAs rated the importance of the lectures significantly higher than MDs (Tables 6, 7). The lectures were rated of “very high importance” or “high importance” by 90.0% of RNs and 91.4% of SCHAs, and, by 87.1% of the MDs.

- H. Evaluate the importance of the links in the program. I. Evaluate the importance of the exercises and the feedback in the program. J. Evaluate the importance of the physical meeting(s) in the program.

Correspondingly, the importance of the IT-links (H), the exercises and feedback from the tutor (I) and of the physical meetings (J) were considered of “very high importance” to “high importance” across professions, by 43.8, 83.9 and 91.7%, respectively. The importance of the links in the program was rated significantly higher by the SHCAs than by MDs and RNs. The importance of the exercises and the feedback was rated significantly higher among SHCAs than other professions (Tables 6, 7). Although all the professions rated the physical meeting(s) in the program of “very high importance” or “high importance”, with no statistical significance across professions, the relative ranking was highest for SHCAs (86.6%), followed by RNs (82.5%) and MDs (72.5%) (Tables 6, 7).

#### Drop-out rates

From January 1, 2005 to December 31, 2014, the overall drop-out rate, across *Educational Fields* and professions, was 499/4693 (10.6%). The drop-out rates were significantly lower (*P* < 0.0001; Chi-square tests) for *Programs* in Chemotherapy (2.9%) and Oncology (3.6%), compared to Radiotherapy (17.5%) and Symptom Therapy (18.7%). The overall drop-out rates were significantly higher (*P* < 0.0001; Chi-square tests) for MDs (18.7%), and, SHCAs (21.4%), compared to RNs (4.3%) and RTTs (8.6%). The lowest drop-out rate was seen for RNs (*P* < 0.0001; Chi-square tests), compared to other professions. Interestingly, linear regression analysis demonstrated a highly significant correlation in the annual increase in overall, relative drop-out rates [r = 0.77 (95% CI 0.27–0.94]; *P* = 0.0095; Pearson].

## Discussion

This study presents nationwide experience with multi-professional, educational E-programs in oncology, during a 10-year period, including nearly 5000 participants. Self-reported outcomes, assessed at completion of the education revealed a high overall contentment, and perceived clinical usefulness of the E-programs, across professions. However, the descriptive design of study does not allow any firm didactic conclusions to be drawn about the E-programs, but the study is at best considered of hypothesis-generating nature, presenting valuable high-volume data for future more scientific rigorous research.

Clinical oncology is an expanding branch of medicine, and does not only include the traditional disciplines as chemotherapy, hormone therapy, immunotherapy and radiotherapy, but also a number of adjuvant specialties, such as palliative medicine, oncological pain management, psychosocial oncology, supportive care and surgical oncology. Educational measures in clinical oncology thus require both multi-disciplinary and multi-professional approaches. The present learning paradigm reflects this well, not only mirrored in the different professions and proficiency records of the trainees, but also in the professional records of the tutors and producers. The educational material presented in our E-programs benefits by the homogenous design across professions and disciplines, facilitating clinical cross-professional and cross-disciplinary collaboration. The statistical significant differences in attitudes and perceived utility of the E-programs between the professions (Table 7), are interesting, indeed, but the authors have not been able to recover any systematic analysis in the literature of differential attitudes towards E-learning, between health care professions. Although very pertinent for detailed didactic, scholarly discussions, these observations await further explorative analyses.

The main advantage of the study are inclusion of a large number of participants (n = 4693) attending a considerable number of E-programs (n = 490), with a study duration of individual educations ranging from days to months (full-time: 0.4–24 weeks). The average drop-out rate of 10.6% was considerably lower than previously described in E-programs, ranging between 25 and 60% [15, 16]. A course retention near 90% is an indirect measure of the contentment and the perceived didactic value of the E-programs and thus is a critical measure of training efficiency. While, the low drop-out rates likely are of multifactorial origin, detailed prospective studies of our E-learning programs are needed to explain these favorable results, particularly regarding the inter-professional differences observed, i.e., significantly lower drop-out rates for RNs and RTTs, compared to MDs and SHCAs. However, the interesting, but disappointing observation of a highly significant linear annual increase in overall, relative drop-out rates probably is likely explained by a rapid expansion of the programs going from 150 trainees 2005, to 700 trainees 2014 (Fig. 2), perhaps indicating an organization in “growing pains.” The hierarchical structure, considered important during the early development of the E-learning programs, was successively replaced with a more horizontal management structure, due to an increased number of programs, trainees, tutors and producers, also reflected in the wider geographical catchment area.

An important aspect in development and designing of E-learning programs is a tendency towards the use of a “richer” medium over time, progressing from text to a graphical platform, and from audio to video recordings, in an attempt to increase the effectiveness of the programs [17, 18]. One study indicated that a “richer” medium content positively correlated with the concentration efforts of the user, but ambiguous results were obtained with the perceived usefulness of the program [17]. Another study observed that the relationship between the “richness” of media choice and the effectiveness was moderated by the learning domain of the program and the learning styles of the user [18]. In the design of our E-learning programs from 2005 to 2014, transitions from audio to video recordings were not implemented to any higher degree since our empirical data indicated that no gain in didactic quality nor trainee satisfaction was obtained by use of a “richer” medium. More studies examining the influence of different media for contents of e-learning programs, regarding the principal outcomes educational efficiency, perceived usefulness, satisfaction scores and cost-efficiency measures, are clearly needed [18].

### Limitations

*First*, an important limitation of this study is that the major outcome, self-reported evaluations only contained data from 2008 to 2015, and, further that these data only comprised 72% of the total number of trainees in this period. Although emphasis on quality assessment from the beginning was considered essential, a number of different evaluation questionnaires were used during the years, impeding the quality of data sampling. Another reason for the incomplete data collection is that a number of programs were locally managed and supervised, and thus, not infrequently, beyond our quality control. *Second*, this retrospective study does not include any control group: a meaningful comparison with other programs, although highly relevant indeed, is therefore not possible at this stage of research. This makes it even more difficult to draw conclusions about the potential advantages of E-learning compared to face-to-face lectures or even computer-based instructions. Comparisons between E-learning and traditional classroom teaching have been evaluated in several studies [19–22], but the published experience in oncology is rather limited [23]. However, the systematic review and meta-analysis by Lahti et al. [9] did not demonstrate any statistical differences between E-learning and traditional learning groups, regarding knowledge, skills or satisfaction. In a recent randomized controlled trial, comparing live lecture, internet-based and computer-based instruction [24], it was observed that even if the interactive Internet-based instruction is a difficult and time-consuming teaching method, it was recommended to integrate the method in medical teaching. Further, in a prospective randomized controlled study [25] including physiotherapy students participating in an oncology course, comparing traditional classroom with E-learning, concluded that the use of E-learning in oncology is a feasible method of teaching. Moreover, Alfieri et al. [10] showed that the use of interactive E-learning for radiation oncology is an effective method to improve the radiologic anatomy knowledge and treatment planning skills of radiation oncology residents. Another advantage, which should be mentioned, is that E-learning may increase the cost-efficiency since the same E-learning program can be transmitted to a larger number of students [26], reducing the demand for a classroom teacher, offering more flexibility [22]. *Third*, only self-reported outcomes, vis-à-vis objective outcomes, are available, and obviously, subjective responses are vulnerable to a number of biases [27]. The printed questionnaire was a structured interview, administered to the trainees during a physical meeting with the tutor(s) upon completion of the program. The questionnaire was answered independently and anonymously by the trainee, and the scoring sheet was collected manually by the tutor. Acquiescence bias, referred to as “yea”-saying or “nay”-saying, i.e., a stereotype response defined as a tendency to agree with attitude statements, regardless of content [27] may have confounded the outcome. In addition, acquiescence bias is likely augmented by our use of similar categorical rating scales among items not conceptually related. Extreme responding bias, a behavior only selecting anchor values, as well as leniency bias, a behavior projecting social relationships upon the evaluation, could also contribute to the skewed distribution seen in response ratings. On the other hand, it could be argued that the self-reported evaluations were accumulated from a large number of participants, professions, programs, covering different topics and time spans, securing a broad platform of experience. The long-term impact of E-learning on clinical practice has been examined in a number of controlled randomized studies [28–31]. Some of these studies observed improvement in the knowledge, skills and clinical behavior of the personnel [30, 31], while others only demonstrated slight, if any advantage over conventional learning [28, 29]. This is corroborated by two recent systematic reviews which concluded that there was insufficient evidence regarding the effectiveness of E-learning on healthcare professional behavior or patient outcomes [9, 32]. *Fourth*, the learning experiences have not yet been tested in a clinical benchmarking scenario against conventional on-location programs.

## Conclusions

This descriptive study presents high-volume data and covers 10-years of nationwide multi-professional and multidisciplinary experience with oncological E-learning. The consistent high fulfillment ratings in learning objectives, the low drop-out rates and the wide geographical catchment area suggest that E-learning programs with contemporary techniques are feasible and complementary pathways to improve pre- and post-graduate training in oncology. Further, the contemporary demands placed on the health workforce regarding a professional responsibility of maintaining the medical competence in practice, E-learning may play an important role in overcoming this challenge [32].

Finally, these hypothesis-generating data demonstrate that our educational paradigm has been well received across disciplines, professions and proficiency levels. However, it is also evident that prospective, high-volume comparative studies, ascertaining the objective didactic value of the E-programs, are needed.

## Additional file

**Additional file 1.** Individual raw data on groups, chronology, the number of participants, completion rates, evaluation responses, geographical distribution of trainees, ECTS-points and teacher demographics.

## Abbreviations

APRN: advanced practice registered nurses
BAS: basic level
CD: compact disc
ECTS: European Credit Transfer and Accumulation System
EXP: experienced level
HCA: health care assistants
HVE: higher vocational education
IGRT: image-guided radiotherapy
IQR: inter-quartile range
IT: information technology
MCQ: multiple choice questions
MD: medical doctors
RES: residents (MD)
RN: registered nurses
RT: radiotherapy
RTT: radiation therapy technologists
SHCA: social and health care assistants
SPEC: specialists (MD)
VC: vocational clinical training
